# To be or not to be: regulation of restriction–modification systems and other toxin–antitoxin systems

**DOI:** 10.1093/nar/gkt711

**Published:** 2013-08-13

**Authors:** Iwona Mruk, Ichizo Kobayashi

**Affiliations:** ^1^Department of Microbiology, University of Gdansk, Wita Stwosza 59, Gdansk, 80-308, Poland, ^2^Department of Medical Genome Sciences, Graduate School of Frontier Sciences, University of Tokyo, Tokyo 108-8639, Japan and ^3^Institute of Medical Science, University of Tokyo, Tokyo 108-8639, Japan

## Abstract

One of the simplest classes of genes involved in programmed death is that containing the toxin–antitoxin (TA) systems of prokaryotes. These systems are composed of an intracellular toxin and an antitoxin that neutralizes its effect. These systems, now classified into five types, were initially discovered because some of them allow the stable maintenance of mobile genetic elements in a microbial population through postsegregational killing or the death of cells that have lost these systems. Here, we demonstrate parallels between some TA systems and restriction–modification systems (RM systems). RM systems are composed of a restriction enzyme (toxin) and a modification enzyme (antitoxin) and limit the genetic flux between lineages with different epigenetic identities, as defined by sequence-specific DNA methylation. The similarities between these systems include their postsegregational killing and their effects on global gene expression. Both require the finely regulated expression of a toxin and antitoxin. The antitoxin (modification enzyme) or linked protein may act as a transcriptional regulator. A regulatory antisense RNA recently identified in an RM system can be compared with those RNAs in TA systems. This review is intended to generalize the concept of TA systems in studies of stress responses, programmed death, genetic conflict and epigenetics.

## INTRODUCTION

The term ‘programmed death’ is usually associated with multicellular organisms. Under certain conditions, an internal signal induces the death of individual cells as a ‘sacrifice’ to maintain the well-being of the entire organism. Similarly, in a unicellular microbial population, the probability of survival might be improved if some members die to ensure the success of the other members or of the population. Such killing in prokaryotes is sometimes mediated by toxin–antitoxin (TA) modules. Death via TA modules can be triggered when an organism is exposed to an environmental stress, such as nutritional or oxygen limitation, DNA damage, antibiotics or phage infection (1–3).

**Figure 1.**
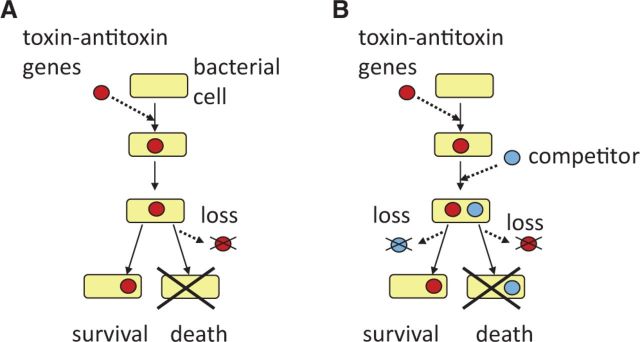
Postsegregational killing or genetic addiction. (**A**) A TA system enters a bacterial cell and establishes itself in the bacterial lineage. Loss of the TA system from the cell triggers the death of its descendant cells. The TA system appears to be stably maintained in the population of viable cells. (**B**) Competitive advantage of TA systems. A cell maintains a TA system on a genetic element. A competitor genetic element enters the cell so that the two genetic elements are both present in the cell, although they are mutually exclusive in the long term. The loss of the competitor element (left) allows the survival of the descendant cell with the TA system, whereas the loss of the element with the TA system (right) leads to the death of the descendant cells, together with the competitor genetic element. The genetic element and the competitor genetic element can be two alleles of a single genomic locus.

Programmed cell death induced by the segregational loss of a genetic element (such as a plasmid) from a cell is called ‘postsegregational killing’ or ‘genetic addiction’. This genetic relationship creates pressure on the host cell to maintain the genetic addiction modules and to pass them on to its descendants (Figure 1A). As long as the modules are transferred, the host remains viable. There are always a certain number of copies of TA systems (TA modules) in the cell, albeit relatively few. However, if the addiction module is lost or displaced by a competing horizontally transferred genetic unit, death is triggered (Figure 1B). The loss of a TA module from a cell leads to the preferential degradation of the antitoxin, which allows the toxin to kill the module-free descendants. This process was first noticed as a mechanism for the stable maintenance of plasmids in microbial populations (3,4). Chromosomally encoded TA modules also act to stabilize linked genes, genomic islands and integrated conjugative elements (5–8).

Cell killing may result from genetic conflict involving several restriction–modification (RM) systems (9,10). RM systems consist of a modification enzyme that methylates a specific DNA sequence in a genome and a restriction endonuclease that cleaves DNA lacking that methylation. These systems thus provide a barrier against genetic flux between different lineages or, more specifically, between lineages with different epigenetic identities, as defined by the RM systems.

As we see later in the text, there is as yet no consensus on the biological significance of TA systems or RM systems. RM and TA systems have many features in common, although no homology has so far been detected between them (11). For example, some RM systems show postsegregational killing (10,12,13). The roles of restriction enzymes are similar to those of the toxins of TA systems, and modification enzymes are similar to their antitoxins. In this review, we may refer to TA systems combined with RM systems as ‘TA systems’ in the broad sense of the term. We may also refer to a restriction enzyme as a ‘toxin’ and a modification enzyme as an ‘antitoxin’.

In light of recent findings, we review several features shared by RM and TA systems. In this comparison, we will focus on the regulation of their gene expression, particularly that involving RNA.

Recently, related reviews have been published that focused on different interests and perspectives: TA systems in the stress response (14); RNA in TA systems (15,16); RM systems in genetic conflict (10); RM systems driving evolution (17); and diverse cellular RM functions (18).

## COMMON THEMES IN THE BIOLOGY OF TA AND RM SYSTEMS

### Classifying TA systems

**Figure 2.**
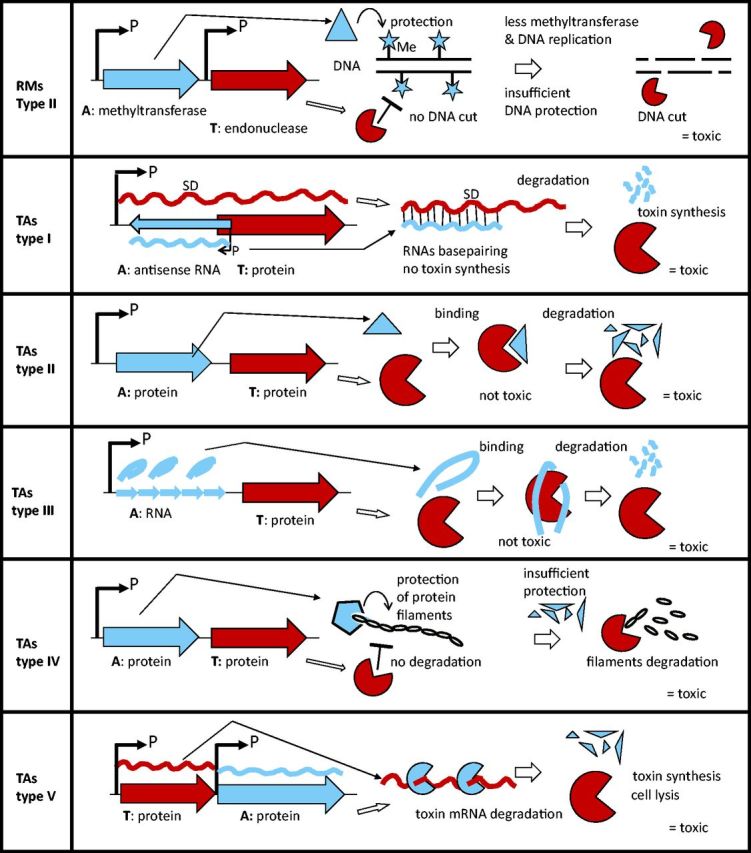
TA systems and Type II RM systems. The toxin is a protein in all cases. The rightmost parts indicate the toxin action after the loss of the genes or an imbalance between the toxin and the antitoxin. Type II RMs have antitoxin modification enzymes that methylate the genomic DNA, protecting it from cleavage by a restriction enzyme (toxin); type I TA has an antisense RNA (the antitoxin) that pairs with the toxin mRNA (19,20); type II TA has a protein antitoxin that binds to the toxin (21,22); type III TA has an antitoxin RNA (not an antisense RNA) that binds to the toxin (23,24); type IV TA has a protein antitoxin that binds to the toxin target (25); and type V TA has a protein antitoxin that cleaves the toxin mRNA (26). A, antitoxin; T, toxin; P, promoter; Me, methyl group on a DNA base; SD, Shine–Dalgarno sequence. Toxins and their genes are shown in dark red; antitoxins and their genes are shown in light blue.

The genetic modules of TA systems are units that produce a functional, stable, toxic protein and its inhibitor, a more labile antitoxin. TA systems are divided into the following types based on the nature of the antitoxin’s inhibitory effect (Figure 2):

- Type I has an antisense RNA as the antitoxin, which pairs with the toxin mRNA (19,20);
- Type II has a protein antitoxin, which neutralizes the toxin’s effect by binding directly to it (21,22);
- Type III has an antitoxin RNA (that is not an antisense RNA), which interacts directly with the toxin protein (23,24);
- Type IV has a protein toxin and a protein antitoxin, which interferes with the binding of the toxin to its target (rather than inhibiting the toxin via direct binding) (25); and
- Type V has a protein antitoxin, which cleaves the toxin mRNA (26).

Our conception of TA systems, presented in this review, has been formed primarily through research into type I and type II systems. Types IV and V have been identified recently, and more members must yet be evaluated. The recent classification of type II TA systems has defined 12 toxin superfamilies and 20 antitoxin superfamilies based on their specific functional capacities to associate with the TA partner and cross-neutralize it (Tables 1 and 2) (22,61). For type I TA systems, small hydrophobic candidate proteins are identified by similarities in their features beyond any sequence similarities (20).

**Table 1.** Toxins in TA systems

Type	TA system	Toxin	Activity	Reference
II	*mazE–maz*	MazF	cleaving mRNA	(27,28)
	*phd–doc*	Doc	binding to ribosomal 30S subunit (blocking translation elongation)	(29)
	*vapB–vapC*	VapC	cleaving initiator tRNA	(30,31,32)
	*ccdA–ccdB*	CcdB	inhibiting DNA gyrase	(33–36)
	*epsilon–zeta*	Zeta	ATP-dependent kinase	(37–40)
	*hipA–hipB*	HipA	serine kinase	(41–43)
III	*toxN–toxI*	ToxN	cleaving mRNA	(23,44)
IV	*cbtA–cbeA*	CbtA	inhibiting cytoskeleton polymerization	(25)
V	*ghoT–ghoS*	GhoT	lyzing cell membrane	(26)

For type I TA systems, see Table 2.

**Table 2.** Type I TA systems

TA system	Host	RNA antitoxin (overlaps mRNA)	Protein toxin	Toxin target	Location	Reference
*hok–sok*	*E. coli*	*sok (5′ end)*	Hok	Cell membrane	Plasmid	(4,45)
*ldrD–rdlD*	*E. coli*	*rdlD* (*5′ end)*	LdrD	Cell membrane	Chromosome	(46)
*istR–tisB*	*E. coli*	*istR-1* (*5′ end)*	TisB	Cell membrane	Chromosome	(47,48)
*symE–symR*	*E. coli*	*symR* (*5′ end)*	SymE	mRNA	Chromosome	(49)
*ibsC–sibC*	*E. coli*	*sibC* (*5′ end)*	IbsC	Cell membrane	Chromosome	(50,51)
*txpA–ratA*	*B. Subtillis*	*ratA* (3*′ end)*	TxpA	Cell membrane	Chromosome (prophage)	(52,53)
*fst*–RNAI–RNAII	*E. faecalis*	RNAII (*3′ end)*	Fst	Cell membrane	Plasmid	(54–58)
*srnB–srnC*	*E. coli*	*srnC* (5*′ end)*	SrnB	Cell membrane*?*	Plasmid	(59)
*pndA–pndB*	*E. coli*	*pndB* (5*′ end)*	PndA	Cell membrane	Plasmid	(59)
*shoB–ohsC*	*E. coli*	*ohsC* (*5′ end)*	ShoB	Cell membrane	Chromosome	(50)
*bsrG*–SR4	*B. subtilis*	SR4 (*3′ end)*	BsrG	Cell membrane	Chromosome (prophage)	(60)

Sometimes, the homologue of a known toxin seems to occur in the absence of a linked gene for a cognate antitoxin homologue in the genome. Likewise, an antitoxin gene homologue can be found without a linked toxin gene. Their products are called ‘solitary toxins’ and ‘solitary antitoxins’, respectively, in this review.

### Classifying RM systems

RM systems are, confusingly enough, also classified into Type I, Type II and Type III. The use of the upper case letter T in the word ‘Type’ is recommended in a new nomenclature system for RM systems (62).

Among the RM systems, a typical Type II system (62), such as EcoRI, carries the restriction enzyme activity and the modification enzyme activity on two separate proteins, as in some TA systems. The Type II RM systems examined show postsegregational killing (see later in the text). Therefore, in this review, we primarily limit our discussion to typical Type II RM systems, calling them simply ‘RM systems’, unless otherwise stated. These RM systems act like type IV TA systems, where the modification enzyme (antitoxin) activity prevents the cleavage of its target by the restriction enzyme (toxin).

The Type I RM systems examined do not display postsegregational killing (63), but they may attack the host chromosome under specific physiological/genetic conditions (9,10,13,64,65), putatively at an arrested replication fork (13). They are composed of three subunits: S (specificity), M (modification) and R (restriction). S and M form a complex with modification activity, whereas S, M and R form a complex with restriction activity. Type III RM systems are composed of a Mod protein and a Res protein. Mod has modification activity, whereas Mod bound to Res has restriction activity (66).

Type IV ‘restriction systems’ (as opposed to ‘RM systems’) consist of a methyl-specific restriction enzyme (Type IV restriction enzyme), all of which cut DNA with moderate methylated-sequence specificity. Their role in programming host death in conflicts between epigenetic DNA methylation systems (10) is discussed later in the text.

A modification enzyme activity is often accompanied by its cognate restriction enzyme activity in a genome. However, a modification activity may be present in the absence of its cognate restriction activity. Such an activity is called a ‘solitary DNA methyltransferase’ in this review. These activities are biologically important, as we see later in the text. Less frequently, a restriction enzyme (a ‘solitary restriction enzyme’) may be present in a genome in the absence of its cognate modification enzyme. The distinction between a complete RM system and a solitary enzyme is not trivial because genes of a single RM system may not be linked to each other (67).

### Abundance and mobility

TA and RM systems are highly abundant in the prokaryotic world (11,20,22,68–73).

Specifically, type II TA systems are found in many bacterial species, with no clear correlation with their lifestyles or genome sizes (22,72). However, many sequenced genomes that contain from several to >60 predicted chromosomally encoded type II TA systems are slow-growing organisms that thrive under nutrient-limiting conditions, such as *Mycobacterium tuberculosis* (30,72). The type III TA systems seem to be more common in Fusobacteria and Firmicutes, and they are slightly less common in Proteobacteria (24). Many RM systems are found in naturally competent bacteria, including *Helicobacter pylori, Neisseria gonorrhoeae, Neisseria meningititis* and *Haemophilus influenzae* (http://tools.neb.com/∼vincze/genomes/). In contrast, small genomes usually lack TA systems, but this could be linked to the scale of the genome and may be biologically irrelevant to the function of the TA systems (74). Moreover, genomes with no or very few TA or RM systems are often those of intracellular host-associated organisms, such as *Mycoplasma*, *Buchnera*, *Treponema* and *Chlamydia.* However, there are exceptions to this: *Mycobacteria* have many TAs, although they are intracellular organisms, whereas *Rickettsia* have many TA systems but no RM systems (18,22,72,75). This trend may suggest that TA or RM systems provide little, if any, selective advantage for those bacteria limited to intracellular growth (72). These bacteria already appear separate from the genetic flux that is the target of RMs and TAs and that allows their acquisition.

Type II TA systems, like Type II RM systems, seem to be widely spread by horizontal gene transfer (22,72,74,46–79), whereas type I TA systems have evolved by lineage-specific duplication (20). The biological significance of this contrast is not completely understood, but there are some clues to it, as we discuss later in the text. In the following paragraphs, we discuss cases in which the domains within a gene represent units of mobility.

RM and TA systems are often found on potentially mobile genetic elements, including genomic islands. RM systems are found on plasmids, prophages, integrons and transposons (68–70). The type I and II TA systems are often located on plasmids, prophages and integrons (70,71,80). Their presence on these elements can be explained by the stabilization of their maintenance by postsegregational killing (8). RM and TA systems are also found in apparently regular chromosomal positions.

Although the mobility of RM and TA systems is often ascribed to their carriage by a known type of mobile genetic element, some RM systems can move unlinked to any mobile element. They themselves appear to be mobile elements. Some RM units seem to insert into the genome with a short targeted duplication, as do some of the classical DNA transposons (81). Like many DNA transposons, they have imperfect inverted repeats at their ends. Some other RM systems can insert into a genome with long and variable (in the order of 100 bp) target duplications (81,82). Movement of RM systems within a genome is often associated with extensive genomic rearrangements (69,81,83). In some RM systems, the individual component genes appear mobile (81).

Type I and Type III RM systems appear less mobile than Type II RM systems in genome comparison (81). However, close examination has revealed that the target-recognition domain within their genes can be a unit of mobility in the following sense (64,84). In Type III RM systems, a specific amino-acid sequence in the target-recognition domain of the modification protein can move between nonorthologous proteins within a species and also beyond species barriers to spread in the bacterial world (84). In the specificity subunit of Type I systems, an amino-acid sequence that recognizes a specific target DNA sequence may move between nonorthologous proteins. An amino acid sequence may even move between two target-recognition domain sites within one protein (64). The likely underlying mechanism of this movement (‘domain movement’) is recombination at shared DNA sequences flanking the two target-recognition domains.

### Postsegregational killing: toxin versus antitoxin

The postsegregational killing by TA systems (in the narrow sense of the word, excluding RM systems) so far examined relies on the difference in stability of the toxin and the antitoxin. (Parenthetically, some antitoxins have a significant half-life that does not place them in the category of labile proteins.) Cells that do not receive a TA plasmid during cell division are killed by the amount of toxin remaining because they lack the protection of the antitoxin (21,85).

The difference in stability between the toxin and the antitoxin in type II TA systems has been shown to result from the susceptibility of the antitoxin to proteolytic degradation. A number of antitoxins are sensitive to degradation by Lon, ClpPX or ClpPA protease. For example, HipB antitoxin is stabilized in the absence of Lon *in vivo* and degraded by Lon *in vitro*. Under normal growth conditions, HipB neutralizes HipA toxin and represses the transcription of the *hipBA* operon (41,86,87). However, when no new HipB is produced or Lon activity is elevated, HipB turnover results in free HipA (41). A chaperon may interact with the antitoxin to prevent its aggregation and protect it from degradation (88). What starts the whole degradation cascade of the antitoxin protein is not at all clear in any of the TA systems.

In contrast, postsegregational killing by Type II RM systems is expected to operate with no difference in stability between the modification enzyme and the restriction enzyme. After loss of the RM genes, the modification enzyme is diluted by cell division, leading to the exposure of unmethylated recognition sites on newly replicated chromosomes. This will result in DNA cleavage by the restriction enzyme activity remaining and cell death (89). The R and M proteins of the EcoRI system are similar in their metabolic stability (90). However, there may be selection for the instability of the modification enzyme to ensure stronger postsegregational killing. (Here, the unit of selection is the RM system, as opposed to the entire genome.) A mutation in the M protein of EcoRII makes this protein unstable and enhances the postsegregational killing by this RM system (91).

### Actions of toxins

Examples of toxins are listed in Tables 1 and 2. Their classification into superfamilies has been proposed (22). It must be remembered that the overproduction of a toxin will not always result in cell death, but may lead instead to cell stasis. For example, cells with elevated RelE or MazF toxin remain viable but in stasis, and can be rescued by the subsequent induction of the cognate antitoxin (92).

In most type II TA systems, the toxin inhibits translation (61) (31) (93). Some of these toxins are highly potent endoribonucleases that cleave cellular mRNA at specific sequences. Therefore, they are called mRNA interferases (94,95). Some toxins of type II TA systems affect DNA replication by blocking DNA gyrase (33–36,96).

In the type II TA system *epsilon–zeta*, the zeta toxin is an ATP-dependent kinase that inhibits peptidoglycan synthesis. It phosphorylates uridine diphosphate-*N*-acetylglucosamine, a peptidoglycan precursor, so that phosphoenolpyruvate cannot be added subsequently. The resulting phosphorylated form also inhibits this addition (37–39). HipA toxin is a phosphatidylinositol/protein kinase and shows serine kinase activity that autophosphorylates it *in vitro* and *in vivo* (42,43). When the expression of the toxin is elevated, a phosphorylation signal is transduced and cell-wall synthesis is impaired, which leads to cell lysis. Its pleiotropic effects include the inhibition of DNA replication, transcription and translation (40,97). The LetA–LetS system and related TA systems carry serine-protease-like toxins and AAA-ATPase-like antitoxins (98).

In contrast, most of the toxins of the type I and type V TA systems, such as TisB and GhoT, target the inner membrane (26,47,99–101) (Table 2). They inhibit ATP synthesis by depleting the proton motive force, leading to dramatic RNA decay, thus halting protein synthesis (47). However, SymE toxin is thought to be a ribonuclease (49). The Ldr toxin (type I) contributes to nucleoid condensation (46); the Fst toxin (type I) targets the cell membrane, but at lower levels, it also affects chromosomal segregation and cell division (102). A type IV toxin, CbtA, binds to cytoskeletal proteins, MreB and FtsZ, and inhibits their polymerization, resulting in the loss of cell shape and polarity, incorrect cell division and finally death (25).

Some TA systems induce the SOS response, a stress response triggered by DNA damage with RecA and LexA regulators. The actions of the type II TA systems DinJ–YafQ, YafN–YafO and ParE homologues (103–105) and of the type I TA systems (*symE–symR*, *tisB–istR1*) are, in turn, affected by the SOS response (47,49,48,106).

All toxins of the Type II RM systems (Type II restriction enzymes) so far examined are highly sequence-specific DNA endonucleases (78). Recent studies have shown that in some cases, cleavage of DNA–RNA hybrids can be achieved *in vitro* (107), although its significance *in vivo* remains unclear.

The induction of the SOS response during postsegregational killing by Type II RM systems (103) and during unbalanced RM activities (104) is consistent with the fact that bacterial cells die when their genomes are cleaved. Cells form filaments and division is prevented. The RecBCD/RecA machinery can repair DNA damage to some extent to allow survival (108).

### Action of protein antitoxins

More labile antitoxins must hold more stable toxins in check. In type II TA systems, such as Kid–Kis and MazF–MazE, the direct binding of the antitoxin to specific domains of the protein toxin, forming an oligomeric complex, inhibits the toxin activity (109). A toxin may have multiple antitoxin-binding domains with different binding affinities. Therefore, multiple protein complexes with different T and A stoichiometries are possible, such as T2:A2 and T2:A2:T2 (109,110).

In contrast, the type IV antitoxin protein YeeU (CbeA) does not form a complex with the toxin CbtA. Instead, YeeU binds directly to the targets of the cognate toxin, the cell filament-producing proteins MreB and FtsZ. YeeU binding stabilizes protein bundling and helps their polymerization into filaments. This process is inhibited by the CbtA toxin in the absence of antitoxin (25). This antitoxin action is somewhat similar to the action of the antitoxin modification enzyme in RM systems (of Type II), although the latter protects the target (DNA) by chemical modification (methylation).

The antitoxin of one type V TA system, GhoS, is a sequence-specific endoribonuclease that cleaves the mRNA of its cognate toxin, GhoT, preventing its translation (26). In this activity, the GhoS antitoxin resembles the toxins of many type II TA systems, which are the mRNA interferases (94).

### Effects of antitoxins on global gene expression

The effects of antitoxins on their own TA or RM systems will be discussed in later sections. Here, we discuss their effects on global gene expression.

The antitoxin of the *Escherichia coli* MqsR–MqsA TA system directly represses the transcription of the gene encoding RpoS, the stationary-phase sigma factor and the master stress regulator (111). Furthermore, the degradation of the antitoxin during stress leads to a switch from the high-motility state to the low-motility state (leading to biofilm formation) (111). Similarly, the antitoxin DinJ of the YafQ–DinJ TA complex in *E. coli* reduces RpoS levels by an indirect mechanism (112). DinJ represses the cold-shock protein CspE, which boosts the translation of *rpoS* mRNA (112,113).

These TA systems are similar to the prototype type V TA system *ghoS–ghoT* and may be regarded as type V. Their action is similar to the action of RNA antitoxins of the type I TA systems, as described later in the text.

RM systems affect the global gene expression of a genome (12). Each of the multiple DNA methyltransferases methylates many copies of a specific recognition sequence in the genome and they together define a specific methylome (or a series of related methylomes). Each of these methylation events may affect nearby gene expression. Overall, they may define a specific transcriptome/proteome.

Methylation by Type III RM systems controls the expression of a group of genes (‘phasevarion’). Differential methylation places certain genes in an ON or OFF state, effectively generating two distinct cell types with two distinct phenotypes (114). Phasevarions are associated with lateral gene transfer, heat shock protein production, virulence factors, motility and colonization in *Neisseria*, *Helicobacter* and other pathogenic bacteria (114,115).

Solitary DNA methyltransferases have been studied extensively with respect to global gene expression (116–118). DNA adenine methylation by Dam (5′(-GmATC) affects the expression of several genes (99116–101118), and is required for the virulence of *Salmonella*, *Haemophilus*, *Yersinia*, *Vibrio* and pathogenic *E. coli* (118). It also acts to coordinate DNA replication and the cell cycle and for template-strand choice during DNA mismatch repair. The M.CcrII DNA methyltransferase [5′-GmANTC (N = A, C, G, or T)] regulates the cell cycle of *Caulobacter crescentus* (119). Methylation by Dcm (5′-Cm5CWGG [W = A or T]) affects the expression of genes in the stationary phase of *E. coli*. Its Dcm-defective mutants show increased expression of the stress response sigma factor, RpoS and many of its targets in the stationary phase (120).

The transcriptome changes that occur during postsegregational killing by Type II RM systems were analyzed in *E. coli* (12). The induction of SOS genes and the RpoE regulon was followed by the induction of stress-response genes (including the RpoS regulon, and osmotic-, oxidative- and periplasmic-stress genes), biofilm-related genes and many hitherto uncharacterized genes. Death was accompanied by cell lysis and the release of cellular proteins. Some signal seemed to be transduced from the damaged genome to the cell surface, leading to its disintegration. These transcriptomal changes partly parallel the changes that occur in cells treated with bacteriocidal antibiotics. The RM systems and the bacteriocidal antibiotics may activate a single death program (12). We are not aware of any comparable transcriptome analysis of TA-mediated cell death.

### Defense against bacteriophages

RM systems may block the entry of DNA from a lineage with a different improper epigenetic DNA methylation status. They inhibit bacteriophage infection if the bacteriophage is from such a lineage, whereas they do not, if it is from a lineage of the same epigenetic status. However, if the phage DNA of a different epigenetic status survives the attack by the restriction enzyme through recombination repair (121) or some other process (a phenomenon called ‘escape’), the phage DNA will be modified in the same way as the host DNA. The resulting phage and its progeny carrying the modified DNA can easily infect cells with the RM system. Once an epidemic starts within a bacterial population, the RM system is no longer effective against infection.

Type IV restriction enzymes show coevolution, of the arms-race type, with bacteriophage DNA modification systems (122). However, cells are also equipped with other phage defense mechanisms that can be more effective, such as the acquired RNA-based immune system (‘clustered regularly interspaced short palindromic repeat’) (123) and phage exclusion (abortive infection) (124,125). Phage exclusion is a system of altruistic death that protects the cell against infection: an infected cell promotes its own death to abort phage reproduction, thus preventing its spread within the population. Type IV restriction enzymes (methyl-specific restriction enzymes) may abort phage infection when the phage brings in a new DNA methylation system and starts methylating the host chromosome (125).

The link between TA systems and phage resistance has not yet been explored thoroughly. Phage infection, in most cases, shuts off host gene expression, including that of the TA systems, which favors the activity of more stable toxins, as in postsegregational killing. In this context, TA systems might be considered antiphage (abortive) factors (126). [Although the modification enzyme is prone to proteolysis in some Type II RM systems (91), whether this leads to the restriction of the host chromosome after phage infection is unknown.]

Few direct examples of this activity have so far been reported. One elegant study of a type III TA system suggests a mechanism of wide multiphage resistance that functions as an abortive infection system (24). The toxin protein ToxN, a member of the CcdB/MazF superfamily, induces reversible growth inhibition (23). Interestingly, its cognate antitoxin, ToxI, is the neutralizing RNA for the ToxN toxin, but it does not act as an antisense RNA interacting with the toxin mRNA. Rather, ToxI directly inhibits ToxN or outcompetes ToxN for certain cellular targets (23,44).

Other TA systems examined reduce infection by a single group of phages, such as the activity of the type II TA system MazE–MazF against P1 phage (2) and the type I TA system *hok–sok* against T4 phage (127). Overall, the importance of TA systems in the defense against bacteriophages still requires investigation.

### Selective advantage

The biological roles or selective advantage of programmed death systems, such as phage exclusion, become clear only when we focus on the population level and on individual genes and genetic elements, rather than on the level of individual lineages or the entire genome. A property beneficial to a gene or a set of genes may not be as profitable to the entire genome. It is especially clear that genes are the units of selection when they are mobile with respect to the genome.

An experimental/theoretical work on phage exclusion demonstrated that within the context of a spatial structure (as in a solid medium), cells that practice a suicide strategy win in competition with cells without such a strategy. However, the suicide strategy fails in the absence of a spatial structure (as in a well-mixed liquid culture) (125).

In postsegregational killing, TA systems (and Type II RM systems) program the death of cells that have lost their genes. Postsegregational killing systems on plasmids may have been selected because they benefit the plasmids in environments in which multiple plasmids must compete during horizontal transfer and reproduction (3). Mochizuki *et al.* (128) analyzed the population dynamics of plasmids with analytical methods and computer simulations based on the methods of theoretical ecology. A genetic element (such as a plasmid) with a TA module has an advantage over a competitor genetic element (such as an incompatible plasmid) without a TA module. However, the advantage is limited in a population without a spatial structure. In contrast, in a structured habitat, the TA gene complex can increase in frequency, irrespective of its initial density. Several experiments have addressed the competitive advantage of TA systems for plasmids, but they have been undertaken in the absence of a spatial structure (129,130).

The postsegregational killing process probably occurs in chromosomal genes because chromosomal RM systems are resistant to replacement by an allelic DNA (lacking the restriction site) (131,132) through homologous recombination. This process should be important because a chromosomal allele is frequently replaced by an allele transferred from another lineage in a bacterial species (133). The arguments presented earlier in the text on the advantages of postsegregational killing also apply to the competition between the alleles at any chromosomal locus, which occurs through homologous recombination.

The chromosomally encoded TA units include those on superintegrons, which are involved in the stable maintenance of the superintegrons by minimizing the formation of superintegron-free cells (6,7). In a structured habitat, postsegregational killing may provide an advantage in the competition between two integrons or between an integron and an integron-free allele.

It has been suggested that many chromosomal type II TA systems function in the adjustment of gene expression in response to stress, to maintain overall bacterial fitness (72). This concept is not exclusive to the idea discussed earlier in the text that genes that program death confer a competitive advantage. The stress response and death process may form a continuous spectrum, with death as the final resolution. In support of the stress response hypothesis, some type II TA systems in *M. tuberculosis* are induced by hypoxia or macrophage infection, indicating their ability to adapt to stressful conditions (30).

Researchers have analyzed the distinct phenotypes of *E. coli* strains from which all TA systems have been removed. In one study, the deletion of five TA systems in the *E. coli* K-12 strain generated no difference from the wild-type strain when exposed to stress in competitive experiments, and no correlation between the TA systems and greater bacterial fitness was observed (5). In other studies, removing the type II TA systems reduced the number of bacterial persisters when bacteria entered a state characterized by a high tolerance of antibiotics (48) and/or significant growth defects (63). The persister phenotype is not associated with a single mutation, although in selections from knockout libraries, a reduced persister frequency was associated with defects in a number of global regulators (47).

In contrast, the mild overproduction of certain TA system toxins can make cells more tolerant of multiple antibiotics (43,1,135). Interestingly, in the case of the HipB–HipA system, the combination of two specific mutations within the *hipA* gene (*hipA7* allele) produces a protein that is inactive as a toxin but confers a high-persistence phenotype on *E. coli*. The dormant state of such persistent cells depends on the increased synthesis of (p)ppGpp (136,137).

The abundance of TA systems has also been linked to a high level of virulence in bacteria with small genomes, although no direct evidence has been obtained supporting the idea that TA systems are responsible for the expression of any specific virulence factors (138,139). A type II TA system carried by a uropathogenic *E. coli* strain affects its colonization of the bladder and its survival within the kidney, where it resists nutrient limitation and oxidative and nitrosative stress (140). A possible role of the type II solitary toxin MazF–Mx in the multicellular development of *Myxococcus* has also been examined (141,142).

As discussed earlier in the text, some RM systems, as well as solitary DNA methyltransferases, have specific effects on the transcriptome (12,143). Changes in the sequence specificity of RM systems (64,84) and the resulting changes in the methylome may alter global gene expression. Natural selection of the diverse resulting epigenomes may underlie adaptive evolution (17).

### Interactions between multiple TA (or RM) systems

In general, interference between multiple TA systems in postsegregational killing and in other contexts may occur when (i) the system components, toxins, antitoxins or regulatory elements are similar and/or (ii) the system components have the same target (10,68). The interaction between two or more TA systems has not been explored thoroughly until recently, even though many organisms encode several TA systems in their genomes, and some cross-talk between TA system components is expected (35,109). The recent classification of type II TA systems (see ‘Classifying TA systems’ section earlier in the text) is based on their specific functional capacity to associate with their TA partner and cross-neutralize it (22,61).

Such interference between RM systems is easily recognized because RM systems target specific DNA sequences and can affect cell survival and death in a dramatic way. Indeed, there is clear evidence for this interference, as detailed later in the text.

Two RM systems with the same target sequence cannot ensure their maintenance by postsegregational killing because the loss of one RM system does not lead to the exposure of the target DNA sites, which are protected by methylation by the other RM system. Therefore, in the presence of another RM system, an RM system can be lost or inactivated without cell killing (144). This means that recognition sequence of an RM system defines an incompatibility group.

An interaction can be asymmetric when the relationship between the recognition sequences is inclusive. For example, if an RM system recognizes 5′-CCWGG (W = A or T) and another recognizes 5′-CCNGG (N = A, C, G, or T), the loss of the former RM system does not lead to chromosomal cleavage because the latter system protects the sites of the former system. However, the loss of the latter RM system does lead to chromosomal cleavage because the former RM system cannot protect 5′-CCSGG (S = G or C) (145). In a similar way, a solitary antitoxin (modification enzyme) can attenuate host killing after the loss of an RM system with the same recognition sequence, as demonstrated for the Dcm methyltransferase and the EcoRII systems, which both recognize 5′-CCWGG (W = A or T) (146).

In general, chromosomally encoded TA units and RM systems probably provide such ‘immunity’ to host killing following the loss of TA and RM plasmids (131). Chromosomally encoded TA systems may have evolved because of such antiaddiction or vaccination effects against addictive genetic elements. These TA systems on bacterial chromosomes could, in turn, have driven the evolution of plasmid-encoded TA systems, selecting for toxins that are no longer recognized by the antiaddiction module (147).

The physical interaction between the toxin of one type II TA system and the antitoxin of another has been shown to occur between Ccd and ParD (148). Interestingly, these toxins act on different targets (one inhibits DNA gyrase and the other inhibits translation, as an endoribonuclease) and have distinct protein structures. However, the proteins share sequence similarities in certain functional modules. The TA cross-interaction leads to toxin cross-neutralization.

Unexpectedly, the nature of the interaction does not follow the pattern seen in the toxin and antitoxin within the same TA system (148). This suggests that the toxin neutralization mechanism is relatively broad and might even occur between homologous TA systems in a single genome (paralogous TA systems) that have limited similarity.

Several studies have demonstrated cross-activity between TA systems in postsegregational killing (35,147). When the network of interactions overlaps, some TA units may control the activity of other units, as in the type II TA MqsR–MqsA system, which affects the type V TA system, GhoT–GhoS (149). The MqsR toxin, an endoribonuclease, degrades the *ghoS* mRNA by cleavage at 5′-GCU sites, and the reduced GhoS antitoxin cannot stop the expression of the GhoT toxin (149). In a sense, one TA system may interfere with the action of another TA unit, as long as it finds its specific target on the subject TA.

Recent work has identified a solitary antitoxin, Dmd, encoded by bacteriophage T4. This unusual phage protein acts against antiviral TA systems of the host bacterium. As aforementioned, during T4 infection, the host genomic transcription is shut off, and the host antitoxins are quickly degraded, which leaves the toxins to kill the host and abort phage propagation. Dmd neutralizes the toxin activity of two types of TA systems (RnlA–RnlB and LsoA–LsoB) (126,150). Dmd binds to the ribonuclease toxins, RlnA and LsoA, in place of their cognate antitoxins, RlnB and LsoB, even though it has no sequence similarity to these antitoxins. This association allows the phage to continue its replicative cycle.

The excess toxin of one TA system may trigger a positive feedback loop of transcriptional activation of another TA unit. Such a cascade of interactions may cause bistability in growth and hence population heterogeneity (151). When two RM systems are similar in the mechanisms by which they regulate gene expression, their interaction can activate the toxin to kill cells, as detailed later in the text (152).

## REGULATION: TO BALANCE OR NOT TO BALANCE TOXIN AND ANTITOXIN EFFECTS?

TA and RM systems involve mechanisms that tightly regulate their gene expression to suppress or activate their lethal effects on host bacteria. When a TA or RM system enters a new host bacterial cell, it must avoid killing it. This has been demonstrated experimentally for several RM systems, in which the antitoxin (modification enzyme) must be expressed and protect the chromosome before the production of the toxic endonuclease (153). We are unaware of comparable studies of TA systems. The activities and expression of the components in TA and RM systems must be carefully maintained during the maintenance phase of a system. When a TA or RM gene set is somehow lost from a cell, the toxin attacks a specific target as part of postsegregational killing. More generally, killing or growth arrest occurs when an imbalance between the toxin and its antitoxin is generated.

The regulatory machinery of mobile TA and RM systems is expected to be host independent to bypass differences in host factors that might affect their establishment, maintenance and their attack on the host. Most regulatory mechanisms are studied at the level of transcription. All these types of regulation require fine-tuned transcriptional feedback circuits to keep the toxin/antitoxin activities in balance (154–157), and presumably to exaggerate their imbalance under certain circumstances. Antisense-RNA-mediated regulation is discussed later in the text.

### Specific transcription-regulating proteins

Some RM and TA units comprise a third gene product, whose activity as a transcription factor is dedicated to controlling their expression.

Several Type II RM systems use a C (control) protein to coordinate the genetic switch for the toxic endonuclease. Switching from the OFF state to the ON state ensures that the host cells are viable, but the toxin activity is high enough to protect the cell from invading phages. The C proteins are transcriptional activators/repressors that specifically bind and distort a DNA operator sequence through a helix–turn–helix motif (158,159). The C proteins can temporally control their own expression, the expression of the endonuclease and that of the modification enzyme, or a combination of these (160–162). The gene copy number may affect the C protein’s mode of activation (163). The inactivation of C proteins leads to the loss of toxin functions; therefore, these proteins are essential for postsegregational killing (152). Moreover, the requirement for a C protein can delay the expression of the restriction enzyme during the establishment of an RM system in a new host cell (152,153).

C protein incompatibility is based on the specificity of their targets, which are DNA operators, in a mechanism similar to that involved in plasmid incompatibility groups. The C protein of one incompatibility group present in a host can prevent the establishment of an incoming RM system with a C gene of the same group because the C-protein-mediated induction of the restriction enzyme from the incoming RM system causes the death of the host cell (152).

In a similar way, two regulators of TA systems, Omega in the *epsilon–zeta* system and PaaR in the *paaA–parE* system*,* are DNA-binding proteins with a ribbon–helix–helix DNA-binding motif. However, unlike the C proteins of RM units, they act strictly as transcriptional repressors. Omega protein, as well as C proteins (154,155,164), binds cooperatively to a recognition sequence within its own promoter, with an affinity that depends on the number of repeats and their orientation (165–167). The *omega* and *paaR* genes occur within a similar operon architecture, where the repressor gene precedes the antitoxin and toxin genes (105,167). The polycistronic mRNA for the three proteic products is initiated from a strong repressor promoter (105,167). A separate weak promoter upstream the antitoxin gene also produces a bicistronic mRNA for the TA unit (167). Unlike the C-protein-dependent RM operons, in this operon scheme, the repression of toxicity is not completely dependent on the regulatory protein. The inactivation of the C protein causes the loss of toxin expression and therefore the toxicity of the RM system, whereas attempts to inactivate the *paaR* gene by a mutation in the TA gene context led to cell death (105). This indicates that the negative feedback loop requires a repressor as a key factor in the maintenance of the TA complex.

The C protein affects the expression of the downstream R gene through transcription–translation coupling in one RM system (168), but not in another (164).

### Antitoxin as a transcriptional regulator of RM and TA systems

The effects of antitoxins on global gene expression were discussed earlier in the text. Here, we analyze their effects on the expression of their own systems.

In the second type of transcriptional regulation in the RM systems, a modification enzyme (antitoxin) represses the transcription of its own gene by binding to its operator, which overlaps its promoter, in its helix–turn–helix domain at the N-terminus (169–173). In some cases, the coordinated expression of RM systems depends on the methylation status of the cognate recognition site(s) in the promoter of the modification gene (174,175).

For the Ecl18kI RM system, antitoxin autorepression is accompanied by an additional promoter competition mechanism, which ensures the toxin/antitoxin balance (176). The unit consists of two divergently oriented genes that occur in the order: M gene (leftward)/P_R_ (promoter for the R gene)/P_M_ (promoter for the M gene)/R gene (rightward), with the two promoters facing each other (176). When the modification enzyme occupies its operator in P_M_ and prevents its own transcription from P_M_, it does not affect the interaction of the RNA polymerase with P_R_. However, in the absence of the modification enzyme, the RNA polymerase transcribes from P_M_ but interferes with the open P_R_ promoter complex (176).

In a similar regulatory pattern, all the type II TA operons tested (e.g. *mazE–mazF, yefM–yoeB, ccdA–ccdB*) are autoregulated by the protein antitoxins. The antitoxins by themselves act as transcriptional repressors, binding to the inverted repeats embedded in the main promoter to repress the transcription of the operon (27,177–181). The cognate toxins are regarded as ‘corepressors’ (27,177,178,182,183). However, the TA complex binds cooperatively to the DNA and only represses transcription when the complex components are present in the proper stoichiometric ratio (180). If the molecular ratio of T/A is elevated, the TA complex cannot interact with the promoter and allows derepression (184). Such ‘conditional cooperativity’ may control the levels of toxin and antitoxin, limiting the induction of the toxin (110,180,182,185).

### Antisense RNAs in regulation

Another important mode of toxin expression control relies on antisense RNAs. In prokaryotes, small non-coding RNAs are involved in numerous cellular processes (186–188). The biological significance of antisense RNAs among these has been well studied. They are involved in DNA replication, transcription and translation, and they affect conjugation, bacteriophage multiplication and plasmid maintenance. They function through a variety of mechanisms, such as changing their own conformation, base pairing with other RNAs and interacting with DNA or proteins.

Antisense RNA/mRNA interactions can be divided into two modes based on their origin: (i) from the same DNA sequence and (ii) from different DNA sequences. We only discuss type (i) here.

Eleven prototype type I TA systems with an antisense RNA as the antitoxin have been characterized (Table 2). Many of these have orthologues in related sequenced genomes (20,189), whereas some have homologues (paralogues) on plasmids or chromosomes. Their antisense RNAs are usually <80 nt long, with some exceptions of ∼200 nt long (19).

**Figure 3.**
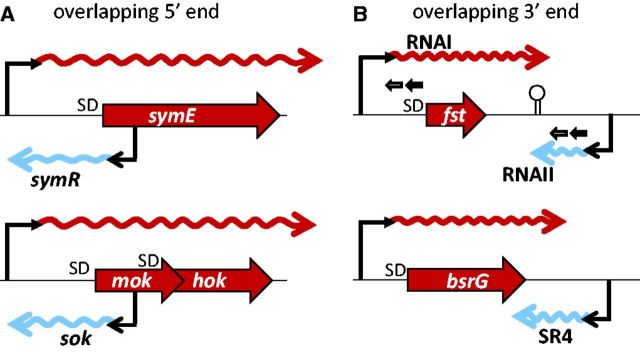
Organization of type I TA systems. A region of the sense mRNA (rightward wavy line) encoding a protein toxin overlaps the coding region of an antitoxin antisense RNA (leftward wavy line) at their 5′ ends (**A**) or 3′ ends (**B**). A bar bent rightward above the gene represents a promoter, and a bar bent leftward beneath the gene represents a reverse promoter. In the *fst*-RNAI–RNAII system, the direct repeats and stem–loop structure are indicated. The figures are not to scale. Modified from: *symE–symR* (49), *hok–sok* (106), *fst*-RNAI–RNAII (54) and *bsrG*–SR4 (60). SD, Shine–Dalgarno sequence.

The regulation of gene expression in type I TA systems is not yet fully understood, but two general schemes of action are so far known: (i) the antisense RNA overlaps the 5′ end of the toxin-encoding mRNA, and the regulation is associated with a translational block as a major contributor; (ii) the antisense RNA overlaps the 3′ end of the toxin mRNA and mRNA degradation is a major factor in the regulatory mechanism (Figure 3). However, in each case, both these contributing factors (translational block and mRNA degradation) act with additional input from proteases and other known and as yet unknown elements.

### Antisense RNA in type I TA systems: pairing with the 5′ region of the toxin mRNA

The *symE–symR* TA unit illustrates a general scheme of regulation, in which the antisense RNA overlaps the 5′ end of the sense toxin RNA (Figure 3A, upper) (15,19,106,50). The antisense RNA (*symR*) produced from a reverse promoter within the toxin gene and the long leader region of the mRNA for the SymE toxin are complementary. The formation of an RNA duplex, in which the 5′ end of the antisense RNA covers the ribosome-binding site (Shine–Dalgarno sequence) of the toxin mRNA, has a strong negative effect on toxin translation (15,49). *SymE* transcription is also repressed by LexA, the binding site of which overlaps the *symE* promoter sequence (49). The toxin is also a target for degradation by the Lon protease (49).

In the *mok–hok–sok* TA family (Figure 3A, lower), the antisense RNA overlaps a gene (*mok*) upstream from the toxin gene (*hok*) in the same orientation and controls the translation of the toxin (Hok) through transcription/translation coupling (190). A strong promoter controls the production of the unstable Sok RNA, which hybridizes with the Mok mRNA and blocks its translation. A weak promoter controls the production of the long highly stable *mok–hok* mRNA (45). This folded mRNA forms of a complex with the Sok RNA through base pairing, which is, in turn, rapidly cleaved by RNase III (191). Toxicity is neutralized in this way during steady-state cell growth because the excess of Sok RNA over *mok–hok* mRNA is maintained (45). However, in cells that do not inherit the *hok–sok* system on a plasmid after cell division, a shortage of labile Sok RNA induces the rapid translation of the toxic Hok protein (4,45,106).

A similar mechanism is thought to operate in the *E. coli* chromosomal *ldrD–rdlD* TA system, which is not homologous to the *hok–sok* system, but it is strikingly similar to it in gene organization (46,106).

### Antisense RNA in type I TA systems: pairing with the 3′ region of the toxin mRNA

In the other mode of regulation of type I TAs, the 3′ end of an antisense RNA and the 3′ end of a toxin mRNA interact (Figure 3B). The best studied TA system with this mode of regulation is located on a plasmid of *Enterococcus faecalis* (Fst toxin) (54,192), but its chromosomal homologues have also been found (193,194). Transcription proceeds from two facing promoters producing (i) the unusually stable sense RNA (mRNA), called RNAI, encoding the 33 amino acid Fst toxin and (ii) the unstable 66 nt antisense RNA, called RNAII, as its antitoxin (Figure 3B upper) (54). RNAII overlaps the sense RNA within a 35 nt region containing a bidirectional stem–loop terminator. The regulation of these two RNAs occurs posttranscriptionally via the interaction of the two complementary direct repeats at their 5′ ends, which allows the stable base pairing of the antisense RNAII and RNAI. This partial duplex sequesters the ribosome-binding site for Fst and blocks toxin translation (55–57). When the plasmid carrying this TA system is lost from a cell, RNAII degradation allows toxin expression from the stable RNAI, and the descendant cells are killed (58,194).

A TA system with similar convergently oriented promoters for two RNAs (BsrG–SR4) was recently identified in a *Bacillus* prophage. The RNAs’ region of overlap is more extended than in the *fst*–RNAI–RNAII system and encompasses 123 bp (Figure 3B, lower). Unlike the Fst TA system discussed earlier in the text, the SR4 antisense RNA primarily promotes the degradation of the BsrG toxin mRNA by targeting the duplex RNA to RNase III. However, the conformational changes in the two interacting RNAs may also interfere with ribosomal access to the ribosome-binding site of the toxin, although no repeats have been detected in their sequences (195).

### Regulation of RM systems by reverse promoters and antisense RNAs

**Figure 4.**
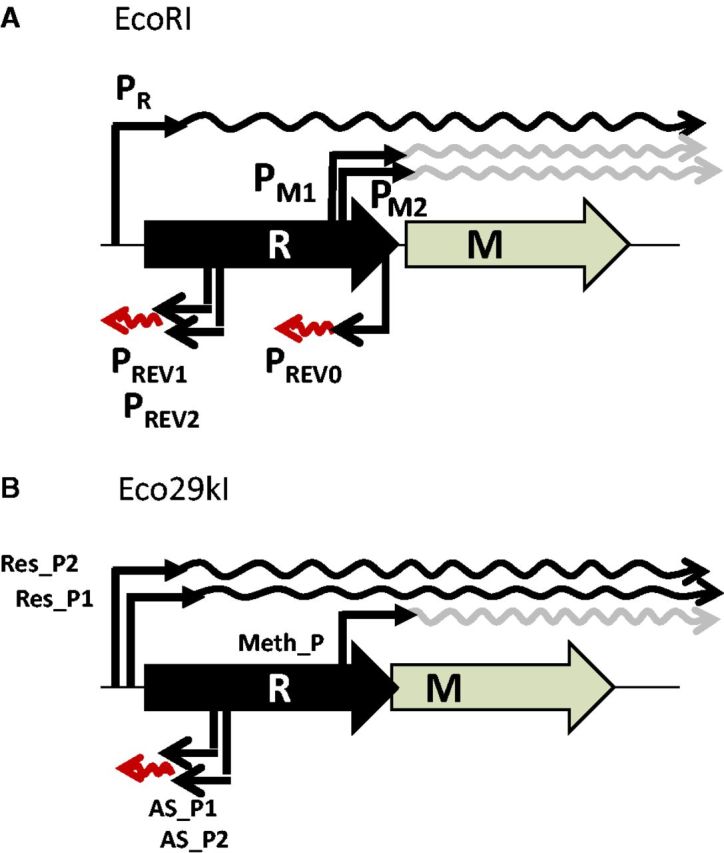
Organization of RM systems with antisense RNA. The promoters (bent bars) and the mRNAs (rightward wavy lines) generated from them are shown above the restriction (R) and modification (M) genes, and the reverse promoters and the antisense RNAs are shown beneath them. A black wavy line represents a bicistronic mRNA, whereas a gray line represents the mRNA for a modification enzyme. A leftward wavy line indicates an antisense RNA. In (**A**), the transcription from P_REV0_ terminates at P_M1M2_. The termination sites for the antisense RNAs from P_REV1_/P_REV2_ and AS_P1/AS_P2 are unknown. The figures are not to scale.

Antisense RNAs have been demonstrated in two Type II RM systems, EcoRI and Eco29I (Figure 4), in which two genes are organized in an operon, with the restriction endonuclease gene preceding the modification enzyme gene. They share a common gene regulation pattern: the promoter upstream from the endonuclease gene drives a long bicistronic mRNA, whereas the modification enzyme can be separately expressed from the downstream P_M_ promoter within the restriction gene.

In the EcoRI transcription unit (Figure 4A), at least six promoters (including two tandem pairs) have been identified experimentally. (Each of the tandem promoter pairs may be regarded as one composite promoter.) Each of the two sense promoters are facing with a reverse promoter within the endonuclease-encoding region: P_R_ versus P_REV1,REV2_ and P_M1,M2_ versus P_REV0_. The intrinsic strength of the promoters, as measured by gene fusion, is in the order P_REV0_ > P_REV1,2_ ≥ P_M1,2_ > P_R_ (196–198). The reverse promoters, P_REV0_ and P_REV1,REV2_, have a strong negative effect on the upstream convergent promoters, P_M1,M2_ and P_R_, respectively (Figure 4A) (197,198). The antisense RNAs from these reverse promoters have been detected (197).

The P_M_-initiated transcription of the modification enzyme is regulated by an 88 nt antisense RNA (Rna0) from a reverse promoter (P_REV0_) (Figure 4A). The two promoters negatively regulate each other (promoter convergence loop) (198). The activation of one promoter above a specific threshold level inactivates the other, further activating the first promoter (199). This feedback loop could represent a bistable switch, similar to those involved in the life cycles of lysogenic bacteriophages (200,201).

If the strength of the reverse promoter (P_REV0_) is reduced, the potency of the restriction of the incoming bacteriophage increases (198). Moreover, the antisense RNA (Rna0) transcribed from the P_REV0_ reverse promoter and delivered *in trans* alleviates postsegregational killing (198). These data demonstrate the biological significance of this antisense RNA. This RM system appears to share a gene regulation strategy with TA systems, in which an antisense RNA and the 5′ end of a toxin mRNA directly overlap (Figure 3A).

The negative effects of reverse promoters, similar to P_REV1,REV2_ in location, on the upstream, forward promoters were analyzed in Eco29kI (Figure 4B) (157). The inhibitory effect was eliminated by introducing a translation initiation signal (Shine–Dalgarno sequence) downstream from the reverse promoters. These data suggest that the base paring between the antisense RNA and the mRNA enhances their degradation, preventing the initiation of toxin translation (157).

### Evolutionary perspectives and concluding remarks

Evolutionary analyses have suggested that the toxin families and antitoxin families of the type II TA systems originated from distinct ancestors that were assembled multiple times during evolution (24). In RM systems, the restriction enzymes originate from different families (202,203), although the DNA methyltransferases form a distinct family, characterized by a specific fold and multiple motifs (204,205). The TA and RM complexes characterized so far do not share an evolutionary origin, and their protein components lack any homology (11). However, the known TA systems are rapidly increasing in number, and many of the known restriction enzymes belong to an uncharacterized fold (202,206); therefore, we cannot exclude the possibility that some homology will be found between these two systems in the future.

The molecular evolution of the TA and RM systems appears to have been complex, probably reflecting their participation in the evolutionary games that often involve genetic conflict (see later in the text). For example, they are characterized by convergent evolution (as opposed to divergent evolution, discussed earlier in the text) and mimicry (see earlier in the text on Dmd). Several antirestriction proteins encoded by mobile genetic elements mimic double-stranded DNA (207).

As detailed earlier in the text, genomic context analyses and genome comparisons have revealed that type II TA systems and many RM systems are mobile. They tend to cluster in ‘defense islands’ (70), and their mobility and addictive properties may allow them to persist for their own benefit. The actions of TA and RM systems that lead to cell death represent genetic conflicts, as we have discussed in detail. This conflict may occur between the mobile elements and the host or between the mobile elements themselves. In general, such genetic conflicts may provide a force driving for evolution (208). There is evidence that conflicts involving RM systems underlie genomic evolution (10,75,125). Indeed, an RM system has been shown to accelerate experimental bacterial evolution (209).

The gene regulation of these systems may be best understood by comparison with that of mobile genetic elements. The effects of several protein antitoxins of TA systems on global gene expression have been discussed earlier in the text, and RM systems affect the global gene expression from a genome, as mentioned earlier in the text (12,114). Switching the target DNA specificity of RM systems (84) will cause changes in the epigenome (or the methylome) and in the global gene expression pattern. This may alter the adaptive phenotypes. These diverse epigenomes may provide the material for natural selection in adaptive evolution. This ‘epigenetics-driven adaptive evolution’ hypothesis, or the concept of selection from diverse epigenomes, is an alternative to the currently popular hypothesis of adaptive evolution, or the concept of selection from divergent genomes (17). Speciation or similar processes at a smaller scale requires the acquisition of an adaptive phenotype and isolation from the genetic flux. Changes in RM systems can achieve both and may be the prime force in prokaryotic speciation and adaptive evolution. Important questions that remain to be answered are what and how internal/external factors affect RM systems, especially their expression and sequence specificity.

We have provided several lines of evidence for mechanisms common to RM and TA systems. Although RM and TA systems represent two groups lacking a common evolutionary origin, they share similar genetic structures, biological functions, evolutionary effects and gene regulatory mechanisms. Detailed analysis of TA and RM systems with experimental, informatic and evolutionary approaches will lead to a deeper understanding of programmed death, genetic conflict, epigenetics and evolution (73,85,210).

## FUNDING

National Science Center (Poland) [grant 7241/B/P01/2011/40 to I.M.]; Grants-in-Aid for Scientific Research from the Japan Society for the Promotion of Science [JSPS; 21370001 and 25291080]; a Grant-in-Aid for Scientific Research from the Ministry of Education, Culture, Sports, Science and Technology [MEXT; 24113506 and 24119503]; and grants from the Global COE (Center of Excellence) Project of Genome Information Big Bang from MEXT and from the Programme for Promotion of Basic and Applied Researches for Innovations in Bio-oriented Industry [121205003001002100019] from the Bio-oriented Technology Research Advancement Institution (to I.K.). Funding for open access charges: the Programme for Promotion of Basic and Applied Research Activities for Innovative Biosciences from the Bio-oriented Technology Research Advancement Institution.

*Conflict of interest statement*. None declared.
